# Resilience buffers the association between psychotic-like experiences and suicide risk: a prospective study from a non-clinical sample

**DOI:** 10.1186/s12888-024-05491-y

**Published:** 2024-01-08

**Authors:** Julia Karska, Maksymilian Rejek, Błażej Misiak

**Affiliations:** Department of Psychiatry, Wroclaw Medical University, Pasteura 10 Street, Wroclaw, 50-367 Poland

**Keywords:** Psychotic-like experiences, Suicidal ideation, Resilience, Psychopathology, Early intervention

## Abstract

**Background:**

Several studies have reported that psychotic-like experiences are associated with low levels of resilience and increased suicide risk. However, it remains unknown as to whether resilience mediates or moderates the association between psychotic-like experiences and suicide risk. Therefore, in this study, we aimed to explore the moderating and mediating effect of resilience in the association between psychotic-like experiences and suicide risk.

**Methods:**

A total of 1100 non-clinical, young adults (aged 18 – 35 years) with a negative history of psychiatric treatment were enrolled. Participants were recruited by the snowball sampling methodology through advertisements posted in the online platform. They were followed-up for about 7 months. Variables of interest were recorded using self-reports. Psychopathological assessment was conducted using the Prodromal Questionnaire-16, the Patient Health Questionnaire-9, the Generalized Anxiety Disorder-7, the Traumatic Experience Checklist, the Childhood Experience of Care and Abuse Questionnaire, the Cannabis Problems Questionnaire, the Connor-Davidson Resilience Scale-10, and the Mini-International Neuropsychiatric Interview. The STROBE statement guidelines were followed.

**Results:**

The moderation analysis revealed that higher levels of psychotic-like experiences and related distress at baseline were associated with significantly higher suicide risk at the follow-up after adjustment for baseline sociodemographic characteristics, depressive and anxiety symptoms, a history of childhood trauma, and problematic cannabis use. The interaction between follow-up resilience and distress related to baseline psychotic-like experiences was significantly and negatively associated with suicide risk at the follow-up. Specifically, the correlation between the level of distress related to psychotic-like experiences and suicide risk was significant and positive only in participants with lower levels of resilience. This interaction did not reach statistical significance for the baseline level of psychotic-like experiences. No significant mediating effect of the follow-up resilience level in the association between baseline psychotic-like experiences and the follow-up suicide risk was found.

**Conclusions:**

Findings from the present study indicate that resilience might protect against suicide risk in people with psychotic-like experiences. These findings could be applied in the formulation of early intervention strategies aimed at mitigating the risk of suicide. Future studies need to explore the effects of interventions targeting resilience for individuals with psychotic-like experiences.

**Supplementary Information:**

The online version contains supplementary material available at 10.1186/s12888-024-05491-y.

## Introduction

Psychotic-like experiences (PLEs) represent prevalent phenomena, with a median annual prevalence estimated at 7.2% as indicated by a systematic review and meta-analysis [1]. Conventionally, PLEs are defined as subclinical symptoms manifesting in the absence of underlying pathology according to international diagnostic frameworks. Although it has initially been shown that PLEs are a risk factor for overt psychosis, recent studies indicate that PLEs may also serve as indicators for a broader spectrum of psychopathology [2, 3]. To date, various factors have been postulated to predict the transition of PLEs to psychosis, including the level of distress associated with PLEs [4, 5]. Additionally, distress related to PLEs may be influenced by other factors, such as a history of traumatic events and co-occurring depressive symptoms [6].

Accumulating evidence indicates that PLEs increase the risk of suicidal ideation, suicide attempts, and death by suicide [7, 8]. A recent meta-analysis revealed that individuals with PLEs are at over threefold higher risk of engaging in self-injurious behaviors [9]. Also, recurrent PLEs are associated with a higher suicide risk compared to transient PLEs [10]. Moreover, the risk of suicide shows some variability across various subtypes of PLEs. For instance, persecutory ideation, thought control, suspicion, auditory hallucinations, and nihilistic thinking/dissociative experiences have been associated with significantly higher suicide risk compared to other subtypes of PLEs [11]. Finally, there might be a dose–dependent relationship between endorsement of PLEs and the risk of suicide [12–14].

However, there is limited knowledge about processes that impact the association between PLEs and the risk of suicide. Accumulating evidence suggests the association between PLEs and resilience. Resilience is defined as an adaptive response, characterized by positive adjustments in the context of stress or trauma [15]. There is evidence that resilience is more a dynamic process than a static trait [16]. Resilience has been indicated to play a protective role throughout the entire spectrum of psychosis, spanning from PLEs to overt psychosis [17, 18]. The protective model of resilience posits that while risk factors may exert adverse effects on psychological wellbeing and clinical outcomes, resilience serves as a moderator. The interaction between resilience and risk factors has the potential to mitigate negative effects and decrease the likelihood of adverse consequences. For instance, schizotypal personality traits, as an indicator of PLEs, have been observed to exert an indirect impact on psychological distress mediated by resilience [19]. Moreover, resilience has been demonstrated to act as a mediator in the relationship between childhood adversity and PLEs, highlighting the significance of insecure-anxious attachment in personal resilience resources and insecure-avoidant attachment in interpersonal resilience [20–22].

Previous studies have also shown that a higher level of resilience may protect against suicidality. Resilience-conferring factors are theorized to reside in a distinct dimension from risk factors and function to attenuate the influence of risk factors on subsequent suicidality [23, 24]. For instance, a history of childhood trauma has been found to correlate with reduced resilience, which in turn is associated with elevated depressive symptoms and ultimately a higher suicide risk [25]. Consequently, resilience emerges as a protective factor mitigating the risk of suicidal behavior related to childhood trauma [26]. A similar protective effect of resilience was observed in the association of negative self-compassion with suicide risk, as well as in the association of depressive and anxiety symptoms with suicidal ideation [27, 28].

To date, little is known about the role of resilience in the association between PLEs and suicidality. One study revealed that resilience and insomnia play a chain-mediating role in the association between PLEs and suicidal ideation in college students [29]. In turn, another study demonstrated that higher levels of resilience and perceived social support protect against suicidal ideation among secondary school and college students [30]. However, it is important to note that both studies focused on suicidal ideation and did not record other aspects of suicidality. Moreover, both studies did not control for the effects of co-occurring psychopathological symptoms and shared correlates for suicidality, PLEs, and resilience, e.g., a history of childhood trauma and cannabis use. Therefore, in the present study we aimed to address the following research question: “does resilience mediate or moderate the association between PLEs and the risk of suicide after adjustment for co-occurring depressive and anxiety symptoms as well as shared correlates?”.

## Methods

### Participants

Participants were recruited with the use of snowball sampling methodology through advertisements posted in the online platform developed to perform research surveys. This methodology was selected due to its applicability in investigating populations that share common symptoms, studying topics that participants may prefer not to discuss publicly, and its wide usage in public health research. The recruitment procedures were conducted taking into consideration the sociodemographic characteristics of Polish residents as documented in the 2021 report. All of them were enrolled in March, 2023. They were asked to respond to the survey administered by the computer-assisted web interview. There were two inclusion criteria: age between 18 and 35 years, and a negative self-reported history of psychiatric treatment. The follow-up survey was conducted in October, 2023. The STROBE statement guidelines [31] were followed (Supplementary Table 1).

All respondents provided informed consent for participation in the survey. Some findings from this dataset were published previously [32]. The protocol of this study was approved by the Bioethics Committee at Wroclaw Medical University, Wroclaw, Poland (approval number: 99/2023).

### Assessments

#### PLEs

The Prodromal Questionnaire-16 (PQ-16) that has been developed to screen for psychosis risk states [33] was used to measure PLEs at baseline. It is composed of 16 items recording the presence of various PLEs (true-or-false responses) and related distress (measured on a 4-point scale with responses ranging from 0 – no distress to 3 – severe distress). The presence of PLEs and associated distress was assessed for the preceding 4 weeks. Two items of the PQ-16 might refer to depressive and anxiety symptoms (item 1: “I feel uninterested in the things I used to enjoy” and item 7: “I get extremely anxious when meeting people for the first time”). To avoid the overlap with the measures of depressive and anxiety symptoms, responses to these items were not included in calculating the PQ-16 total scores. Therefore, in the present study, the total PQ-16 score ranged between 0 and 14 for the subscale measuring the presence of PLEs as well as between 0 and 42 for the subscale measuring the level of distress associated with PLEs. In our study, the Cronbach’s alpha of the PQ-16 was 0.843 for the subscale measuring the presence of PLEs and 0.869 for the subscale measuring associated distress.

#### Depressive symptoms

The Patient Health Questionnaire-9 (PHQ-9) was administered to control for the levels of baseline depressive symptoms [34]. It measures the severity of depressive symptoms for the preceding 2 weeks using a 4-point scale. Responses are scored between 0 – “not at all” to 3 – “nearly every day”. The total score ranges between 0 and 27. In our study, the Cronbach’s alpha of the PHQ-9 was 0.878.

#### Anxiety symptoms

The Generalized Anxiety Disorder-7 (GAD-7) was used to control for the levels of baseline anxiety symptoms. It includes 7 items that record the level of anxiety symptoms over the period of preceding 2 weeks. Each item is scored on a 4-point scale (responses range between 0 – “not at all” to 3 – “nearly every day”). The total score ranges between 0 and 21. In our study, the Cronbach’s alpha of the GAD-7 was 0.925.

#### A history of childhood trauma

A history of emotional neglect, emotional abuse, bullying, and sexual abuse before the age of 17 years was measured. Emotional neglect and abuse as well as bullying were assessed using three questions from the Traumatic Experience Checklist (TEC) [35]: “When you were a child or a teenager, have you ever felt emotionally neglected (e.g., being left alone, insufficient affection) by your parents, brothers or sisters?”; “When you were a child or a teenager have you ever felt emotionally abused (e.g., being belittled, teased, called names, threatened verbally, or unjustly punished) by your parents, brothers or sisters?”, and “When you were a child or teenager, did you experience psychological violence (e.g., nicknames, teasing) or physical abuse (e.g., jerking, beating) from your peers?”. In turn, three questions from the Childhood Experience of Care and Abuse Questionnaire (CECA.Q) [36] were used to record a history of sexual abuse: “When you were a child or teenager did you have any unwanted sexual experiences?”; “Did anyone force you or persuade you to have sexual intercourse against your wishes before age 17?” and “Can you think of any upsetting sexual experiences before age 17 with a related adult or someone in authority e.g., teacher?”. The same set of questions from the TEC and CECA.Q has also been used by the prior studies [37, 38]. In the present study, we used the total childhood trauma score as the sum of positive responses to these questions. Therefore, the total childhood trauma score ranged between 0 and 6 with higher scores indicating greater exposure to various categories of childhood trauma. The Cronbach’s alpha for the scale measuring a history of childhood trauma was 0.708 in the present study.

#### Problematic cannabis use

Problematic cannabis use was measured by 11 out of 16 questions from the Cannabis Problems Questionnaire (CPQ) [39] referring to the period of preceding 12 months: “Have you tended to smoke more on your own than you used to?”; “Have you been neglecting yourself physically?”; “Have you felt depressed for more than a week?”; “Have you been so depressed you felt like doing away with yourself?”; “Have you given up recreational activities you once enjoyed for smoking?”; “Do you find it hard to get the same enjoyment from your usual interests?”; “Have you felt more antisocial after smoking?”; “Have you worried about getting out of touch with friends or family?”; “Have you been concerned about a lack of motivation?”; “Have you worried about feelings of personal isolation or detachment?” and “Do you usually have a smoke in the morning, to get yourself going?”. All questions are based on yes-or-no responses (scored 1 or 0). In the present study, the total CPQ score ranged between 0 and 14 with higher scores indicating higher levels of problematic cannabis use. In our study, the Cronbach’s alpha of the CPQ was 0.899.

#### Resilience

The Connor-Davidson Resilience Scale-10 (CD-RISC-10) [40] was administered at the follow-up to record the level of resilience. It includes 10 items that are scored on a 5-point Likert-like scale (0 – “never” to 4 – “almost always). The respondents are asked to assess the level of agreement with all items over the period of preceding 1 month. The total CD-RISC-10 score ranges between 0 and 40 with higher scores indicating greater resilience. In our study, the Cronbach’s alpha of the CD-RISC-10 was 0.920.

#### Suicide risk

To assess the risk of suicide at the follow-up, we used the suicidality section of the Mini-International Neuropsychiatric Interview (M.I.N.I.) [41]. It includes 6 questions with yes-or-no responses. Among them, 5 questions refer to the preceding month: “did you think that you would be better off dead or wish you were dead?” (score: 1 point), “did you want to harm yourself or to hurt or injure yourself?” (score: 2 points), “did you think about suicide?” (score: 6 points), “did you have a suicide plan?” (score: 10 points), and “did you attempt suicide?” (score: 10 points). In turn one question records a lifetime history of suicide attempt (“in your lifetime, did you ever make a suicide attempt?”, score: 4 points). The risk of suicide is calculated as the sum of points for responses to all questions (range: 0 – 33). Higher scores indicate a greater risk of suicide. The Cronbach’s alpha of the M.I.N.I. suicidality section was 0.736 in the present study.

### Statistics

**Fig. 1:**
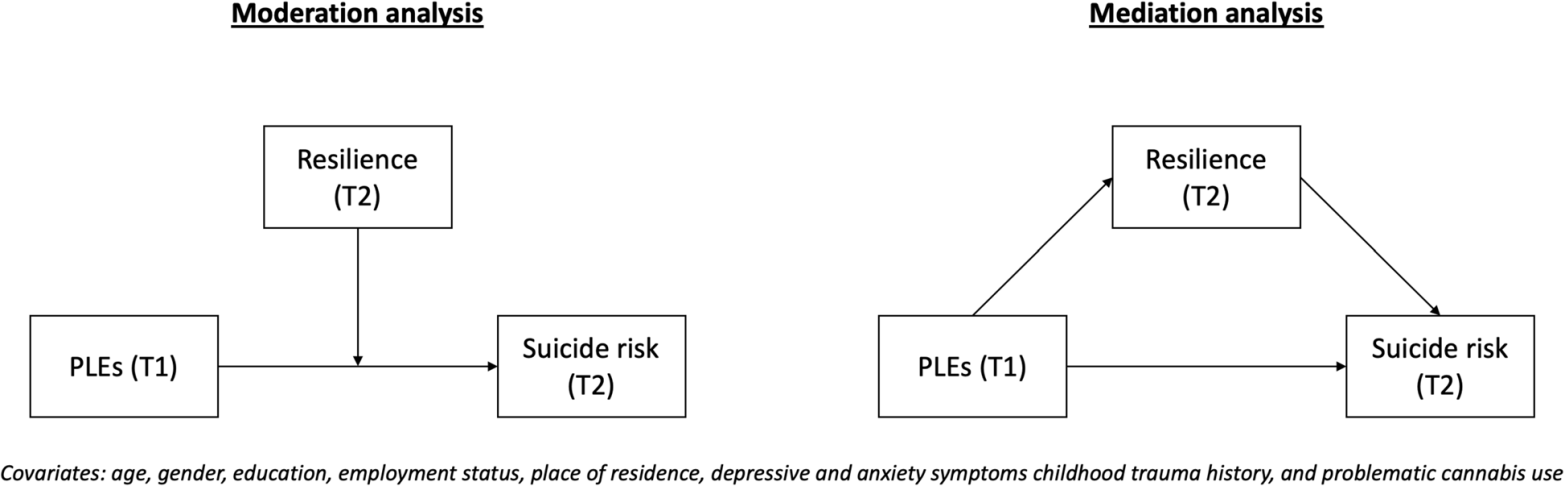
The data analysis plan

Both groups of participants, i.e., participants who completed measurements at both timepoints and those who were lost to follow-up (further referred to as completers and non-completers, respectively) were compared with respect to the general characteristics using t-tests (continuous variables) and the ${x}^{2}$ test (categorical variables). Correlations between continuous variables were tested using the Pearson correlation coefficients. Next, we analyzed as to whether resilience mediates or moderates the association between PLEs and suicide risk using the PROCESS macro models 1 and 4, respectively (Fig. 1). Independent models were analyzed for the presence of PLEs and the level of distress associated with PLEs. The PQ–16 score was included as the predictor, while the M.I.N.I. suicide risk score represented the outcome variable. Covariates were age, gender, the level of education, employment status, place of residence, the levels of depressive and anxiety symptoms, childhood trauma score, and the CPQ score. The Johnson-Neyman technique was applied in order to indicate the range of CD-RISC-10 scores for which the interaction is significant. The interaction was plotted for the 18th, 50th, and 84th percentile values of the CD-RISC-10 score. Results were interpreted as significant if the *p*-value was lower than 0.05. In case of mediation models, results were considered significant if the 95% confidence interval (CI) did not include zero. All analyses were carried out in the SPSS software, version 28. The sample size was established based on a priori power calculations performed in the G*Power [42].

## Results

Baseline assessment was completed by 1100 participants (aged 27.1 ± 5.1 years, 48.6% males) (Table 1). Non-completers reported significantly higher levels of PLEs and depressive symptoms, problematic cannabis use, and exposure to childhood trauma. Completers and non-completers also differed significantly in terms of age, the level of education, occupation, and place of residence. A priori estimations showed that a total of 550 participants are needed to be enrolled in order to detect a small effect size (f^2^ = 0.02) with the power of 80% (α = 0.05, total number of predictors = 12). Due to anticipated dropouts, the target sample size was increased to *n* = 1100.

**Table 1:** General characteristics of the sample

	Total sample (*n* = 1100)	Completers (*n* = 581)	Non-completers (*n* = 519)	*p*
Age, years	27.1 ± 5.1	27.9 ± 5.0	26.1 ± 4.9	** < 0.001**
Gender, males	535 (48.6)	295 (50.8)	240 (46.2)	0.133
Education				
Primary	61 (5.5)	28 (4.8)	33 (6.4)	** < 0.001**
Vocational	89 (8.1)	39 (6.7)	50 (9.6)	
Secondary	553 (50.3)	261 (44.9)	292 (56.3)	
Higher	397 (36.1)	253 (43.6)	144 (27.7)	
Employment status				
Unemployed	164 (14.9)	74 (12.8)	90 (17.3)	** < 0.001**
Part-time	170 (15.4)	69 (11.9)	101 (19.5)	
Student	202 (18.4)	99 (17.0)	103 (19.8)	
Full-time	564 (51.3)	339 (58.3)	225 (43.4)	
Place of residence				
Rural	428 (38.9)	216 (37.2)	212 (40.8)	**0.009**
Urban (up to 100,000 inhabitants)	351 (31.9)	207 (35.6)	144 (27.7)	
Urban (100,000 – 200,000 inhabitants)	101 (9.2)	40 (6.9)	61 (11.8)	
Urban (200,000 – 500,000 inhabitants)	87 (7.9)	46 (7.9)	41 (7.9)	
Urban (> 500,000 inhabitants)	133 (12.1)	72 (12.4)	61 (11.8)	
Resilience, CD-RISC-10	–	20.9 ± 8.2	–	–
Suicide risk, M.I.N.I	–	4.0 ± 7.9	–	–
PLEs, PQ–16 (presence)	4.4 ± 3.6	3.9 ± 3.5	4.9 ± 3.6	** < 0.001**
PLEs, PQ–16 (distress)	13.3 ± 12.7	11.7 ± 12.0	15.1 ± 13.1	** < 0.001**
Anxiety symptoms, GAD–7	7.6 ± 5.5	7.3 ± 5.5	7.9 ± 5.5	0.069
Depressive symptoms, PHQ–9	9.4 ± 6.2	9.0 ± 6.2	9.8 ± 6.1	**0.035**
Childhood trauma score	1.9 ± 1.6	1.7 ± 1.6	2.0 ± 1.6	**0.021**
Problematic cannabis use, CPQ	0.2 ± 1.1	0.1 ± 0.8	0.4 ± 1.4	** < 0.001**

*CD-RISC-10* the Connor-Davidson Resilience Scale-10, *CPQ* the Cannabis Problems Questionnaire, *GAD–7* Generalized Anxiety Disorder–7, *M.I.N.I.* the Mini International Neuropsychiatric Interview, *PHQ–9* the Patient Health Questionnaire–9, *PLEs* psychotic-like experiences, *PQ–16* the Prodromal Questionnaire–16

Significant differences (*p* < 0.05) are marked in bold

**Fig. 2:**
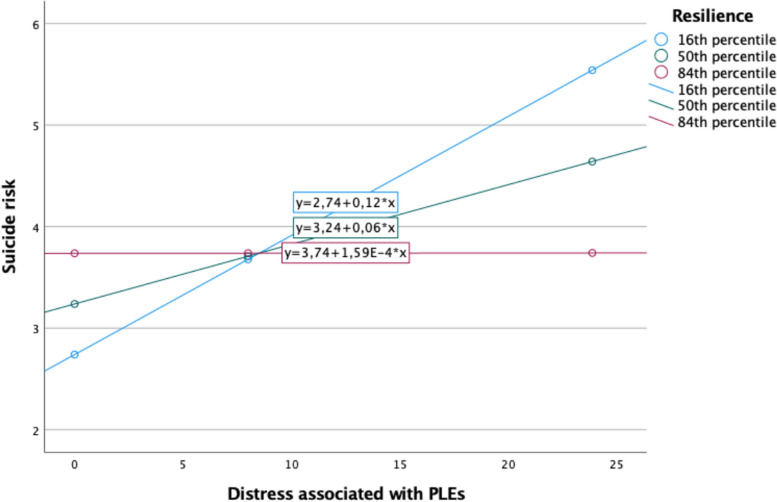
Correlations of the baseline distress related to psychotic-like experiences with the follow-up suicide risk at various levels of follow-up resilience

The majority of correlations between measures assessed in this study were significant (Table 2). However, the correlations between resilience and suicide risk as well as between problematic cannabis use and resilience were not significant. Results of moderation analyses are reported in Table 3. Higher baseline levels of PLEs and related distress, childhood trauma score, and problematic cannabis use were associated with significantly higher suicide risk at the follow-up in both models. The interaction between resilience and distress related to PLEs was significantly and negatively associated with suicide risk at the follow-up (Table 3, Fig. 2). The CD-RISC score defining the Johnson-Neyman region of significance was 24.6 (32.5% participants scored above this cut-off and 67.5% participants scored below this cutoff). Importantly, the interaction between resilience and the level of PLEs was not significantly associated with the risk of suicide (Table 3).

**Table 2:** Correlations between measures used in the present study

	1	2	3	4	5	6	7
1. PLEs – presence (T1)	–						
2. PLEs – distress (T1)	*r* = 0.948^***^	–					
3. Depressive symptoms (T1)	*r* = 0.466^***^	*r* = 0.530^***^	–				
4. Anxiety symptoms (T1)	*r* = 0.460^***^	*r* = 0.521^***^	*r* = 0.521^***^	–			
5. Childhood trauma score (T1)	*r* = 0.353^***^	*r* = 0.361^***^	*r* = 0.369^***^	*r* = 0.344^***^	–		
6. Problematic cannabis use (T1)	*r* = 0.127^***^	*r* = 0.114^***^	*r* = 0.105^***^	*r* = 0.077^*^	*r* = 0.103^***^	–	
7. Resilience (T2)	*r* = –0.140^***^	*r* = –0.137^***^	*r* = –0.275^***^	*r* = –0.231^***^	*r* = –0.134^**^	*r* = –0.049	–
8. Suicide risk (T2)	*r* = 0.185^***^	*r* = 0.209^***^	*r* = 0.192^***^	*r* = 0.199^***^	*r* = 0.206^***^	*r* = 0.140^***^	*r* = –0.076

*PLEs* psychotic-like experiences, *T1* baseline assessment, *T2* follow-up assessment

^*^p < 0.050, ^**^p < 0.010, ^***^p < 0.001

**Table 3:** Results of moderation analyses

Category of PLEs	Independent variable	B	SE	*p*
Presence (R^2^ = 0.096)	PLEs	0.567	0.253	**0.026**
	Depressive symptoms	0.011	0.086	0.898
	Anxiety symptoms	0.141	0.095	0.139
	Resilience	0.062	0.057	0.280
	PLEs × resilience	–0.021	0.011	0.054
	Problematic cannabis use	1.008	0.410	**0.014**
	Childhood trauma score	0.671	0.236	**0.005**
	Age	–0.093	0.073	0.203
	Gender	–0.503	0.691	0.467
	Education	–0.784	0.460	0.089
	Employment status	–0.058	0.333	0.862
	Place of residence	–0.101	0.333	0.676
Distress (R^2^ = 0.099)	PLEs	0.195	0.075	**0.009**
	Depressive symptoms	–0.005	0.088	0.951
	Anxiety symptoms	0.128	0.095	0.179
	Resilience	0.055	0.053	0.300
	PLEs × resilience	–0.007	0.003	**0.035**
	Problematic cannabis use	0.980	0.410	**0.017**
	Childhood trauma score	0.651	0.235	**0.006**
	Age	–0.095	0.073	0.192
	Gender	–0.539	0.690	0.435
	Education	–0.772	0.459	0.094
	Employment status	–0.079	0.332	0.813
	Place of residence	–0.074	0.241	0.758

*PLEs* psychotic-like experiences

Significant effects (*p* < 0.05) are marked in bold

Results of mediation analyses are reported in Supplementary Table 2. There were significant direct effects of PLEs on the risk of suicide after accounting for sociodemographic characteristics, depressive and anxiety symptoms, problematic cannabis use, and the childhood trauma score. However, indirect effects of PLEs on the risk of suicide (through resilience) were not significant.

## Discussion

### Main findings

The present study confirmed that the presence of PLEs and related distress are associated with suicide risk, even after adjustment for sociodemographic characteristics, depressive and anxiety symptoms as well as shared correlates (a history of childhood trauma and problematic cannabis use). These observations are in agreement with a number of previous studies performed in various populations [43, 44]. Simultaneously, our findings indicate that resilience moderates the association between distress related to PLEs and suicide risk. Specifically, we found that the correlation between the level of distress related to PLEs and suicide risk is significant only in individuals with low levels of resilience. This moderating effect did not reach statistical significance for the presence of PLEs. Also, we did not observe that resilience significantly mediates the association between the presence of PLEs or related distress and suicide risk.

Resilience is increasingly being recognized as a complex process covering risk and protective factors that ultimately results in a better outcome than might be expected given the extent or severity of exposure [45]. In this regard, resilience should be perceived as a dynamic process showing considerable variability between individuals across time. Given that resilience shows some extent of within person variability, it might serve as the target for therapeutic interventions. A recent systematic review demonstrated that resilience is inversely associated with PLEs [46]. This correlation was observed by 73% of studies included in a systematic review.

The moderating effect of resilience was found for distress related to PLEs but not their presence. This observation warrants further commentary. Importantly, the potential clinical relevance of distress related to PLEs has been observed by previous studies. For instance, it has been reported that distress might serve as a partial mediator and moderator in the association of PLEs with suicidal ideation and behaviors [11]. Another study demonstrated that PLEs predict suicidal attempts among adolescents only in case of co-occurring distress [12]. Furthermore, there is evidence that individuals with distressing PLEs might be at a higher risk of most unfavorable mental health outcomes compared to those with non-distressing PLEs [47].

Our findings showing that a higher level of resilience serves as a protective factor against suicide in people with PLEs is consistent with those from the recent study performed in college students during the COVID-19 pandemic [30]. The authors found that better resilience and greater social support protect against suicidal ideation in students with PLEs. However, this observation is not in agreement with findings from another study performed in secondary school and college students. The authors found that higher levels of resilience and insomnia act as mediators in a chain-mediating mechanism between PLEs and suicidal ideation [29]. It is essential to note that the studies we compared ours to did not control for shared correlates, such as substance use and a history of childhood trauma [29, 30, 46]. Additionally, these studies were limited to the assessment of suicidal ideation.

### Limitations

There are some limitations of the present study. First, the sample size was not large and our analysis of data was limited to only two time points with a relatively short follow-up period. Second, individuals who were lost to follow-up differed significantly in terms of sociodemographic characteristics, psychopathological symptoms, exposure to childhood trauma, and problematic cannabis use. Third, the level of resilience and suicide risk were assessed only at the follow-up. Fourth, it is worth noting that participants who were lost to follow-up showed heightened vulnerability and increased levels of risk factors for suicide investigated in the present study. Notably, we did not record reasons of attrition. It might be speculated that due to higher levels of psychopathological symptoms and risk factors for mental disorders (i.e., cannabis use and a history of childhood trauma), non-completers required treatment or developed outcomes related to suicidality, and thus were not able to participate in the follow-up assessment. Fifth, the clinical validation of PLEs and other psychopathological symptoms was not performed. However, it should be noted that even self-reported PLEs revealed to be false-positive findings might predict unfavorable mental health outcomes [48, 49]. At this point, it is also important to note a lack of assessment of suicide risk using the Columbia-Suicide Severity Rating Scale that is now perceived as the gold standard tool [50]. However, it was not included as it requires in-person assessment by trained clinicians. Sixth, participant selection through advertisements posted in the online platform developed to perform research surveys may be related to the selection bias or a voluntary bias. Data were self-reported by participants through self-administered questionnaires, with no evaluator validation of adherence to inclusion criteria or verification of provided information. Nevertheless, the sampling methodology with the use of online surveys is particularly useful in case of studies addressing sensitive topics as they provide anonymity [51]. Furthermore, measurements used in the present study were validated and wiedely used in psychiatry. Finally, it is important to note that more complex mechanisms, not assessed in the present study, may underlie the protective effect of resilience on the association between PLEs and suicide risk. For instance, it has been shown that resilience moderates the association between PLEs and sleep disturbance [52]. In turn, our group also demonstrated that PLEs are associated with suicidal ideation only in individuals with higher levels of insomnia [53].

### Implications

Our findings hold some promise to develop interventions aimed at improving resilience for individuals who report distressing PLEs. Indeed, it has been shown that a variety of psychotherapeutic interventions might improve resilience in non-clinical populations [54]. It has also been demonstrated that promoting resilience may reduce suicide risk in various populations (for review see [55]). Moreover, there is evidence from the randomized controlled trial performed in college students with mildly elevated depressive symptoms and PLEs that resilience training might improve the levels of psychopathology and psychological wellbeing [56]. Specifically, the authors found that compared to the waitlist controls, participants assigned to this intervention reported a significant reduction in the levels of PLEs and associated distress, depressive and anxiety symptoms as well as a significant improvement of mindfulness, self-compassion, positive affect, and resilience.

## Conclusions

In sum, our findings indicate that better resilience might serve as a protective factor against suicide risk in people with distressing PLEs. Our findings indicate the potential for formulating early interventions specifically targeting individuals susceptible to psychiatric morbidity, particularly those with PLEs and increased suicide risk. The practical applications encompass a spectrum of intervention strategies, prevention programs, mental health support initiatives, and public health efforts aimed at fostering resilience and reducing the incidence of psychiatric morbidity and suicide risk across diverse populations. Future studies with longer follow-up periods and in-person clinical assessment of participants with standardized interviews are warranted and need to investigate the moderating effect of resilience on the association between PLEs and other mental health outcomes. Moreover, it is further needed to develop therapeutic interventions that aim to improve resilience in people with PLEs.

### Supplementary Information

**Additional file 1: Supplementary Table 1.** The STROBE checklist. **Supplementary Table 2.** Results of mediation analyses.

## Data Availability

No datasets were generated or analysed during the current study.
